# (*R*_p_)-1-{(*R*)-(Dimethyl­amino)[2-(diphenyl­phosphan­yl)phen­yl]methyl}-2-(diphenyl­phosphan­yl)ferrocene chloro­form solvate

**DOI:** 10.1107/S160053680803955X

**Published:** 2008-11-29

**Authors:** Jan W. Bats, Andreas Rivas Nass, Angelino Doppiu, Ralf Karch, A. Stephen K. Hashmi

**Affiliations:** a Institut für Organische Chemie, Universität Frankfurt, Max-von-Laue-Strasse 7, D-60438 Frankfurt am Main, Germany; b Umicore AG & Co KG, Strategic Research and Development, Precious Metals Chemistry, Rodenbacher Chaussee 4, D-63457 Hanau, Germany; c Organisch-Chemisches Institut, Universität Heidelberg, Im Neuenheimer Feld 270, D-69120 Heidelberg, Germany

## Abstract

The absolute configuration of the title mol­ecule, [Fe(C_5_H_5_)(C_38_H_34_NP_2_)]·CHCl_3_, is *R*,*R*_p_. The mol­ecular structure is similar to the structure of the solvent-free compound [Fukuzawa, Yamamoto & Kikuchi (2007 ▶). *J. Org. Chem*. **72**, 1514–1517], but some torsion angles about the P—C_phen­yl_ bonds differ by up to 25°. The P atoms and the N atom have a distorted trigonal-pyramidal geometry. The chloro­form solvate group donates a C—H⋯π bond to the central benzene ring and is also involved in six inter­molecular C—H⋯Cl contacts with H⋯Cl distances between 2.96 and 3.13 Å.

## Related literature

The crystal structure of the solvent-free compound has been reported by Fukuzawa, Yamamoto & Kikuchi (2007 ▶) and the structures of related mol­ecules by Ireland *et al.* (1999 ▶) and Bats *et al.* (2008 ▶). For the synthesis of related compounds, see: Ireland *et al.* (2002 ▶); Fukuzawa, Yamamoto, Hosaka & Kikuchi (2007 ▶). For the stereochemistry of taniaphos ligands, see: Ireland *et al.* (2008 ▶). 
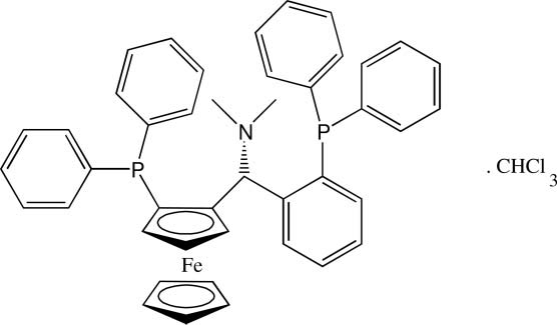


## Experimental

### 

#### Crystal data

[Fe(C_5_H_5_)(C_38_H_34_NP_2_)]·CHCl_3_
*M* *_r_* = 806.91
Orthorhombic, 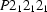

*a* = 10.6051 (11) Å
*b* = 11.8922 (10) Å
*c* = 30.625 (3) Å
*V* = 3862.3 (6) Å^3^
*Z* = 4
Mo *K*α radiation
μ = 0.71 mm^−1^
*T* = 163 (2) K
0.60 × 0.40 × 0.37 mm

#### Data collection

Siemens SMART 1K diffractometer
Absorption correction: numerical (*SHELXTL*; Sheldrick, 2008 ▶) *T*_min_ = 0.664, *T*_max_ = 0.786
59511 measured reflections
11202 independent reflections
9851 reflections with *I* > 2σ(*I*)
*R*_int_ = 0.061
1004 standard reflections frequency: 1200 min intensity decay: none

#### Refinement

*R*[*F* ^2^ > 2σ(*F* ^2^)] = 0.039
*wR*(*F* ^2^) = 0.088
*S* = 1.18
11202 reflections
462 parameters
H-atom parameters constrained
Δρ_max_ = 0.50 e Å^−3^
Δρ_min_ = −0.39 e Å^−3^
Absolute structure: Flack (1983 ▶), 4811 Friedel pairs
Flack parameter: −0.021 (11)

### 

Data collection: *SMART* (Siemens, 1995 ▶); cell refinement: *SMART*; data reduction: *SAINT* (Siemens, 1995 ▶); program(s) used to solve structure: *SHELXS97* (Sheldrick, 2008 ▶); program(s) used to refine structure: *SHELXL97* (Sheldrick, 2008 ▶); molecular graphics: *SHELXTL* (Sheldrick, 2008 ▶); software used to prepare material for publication: *SHELXL97*.

## Supplementary Material

Crystal structure: contains datablocks global, I. DOI: 10.1107/S160053680803955X/nc2126sup1.cif

Structure factors: contains datablocks I. DOI: 10.1107/S160053680803955X/nc2126Isup2.hkl

Additional supplementary materials:  crystallographic information; 3D view; checkCIF report

## Figures and Tables

**Fig. 1.**
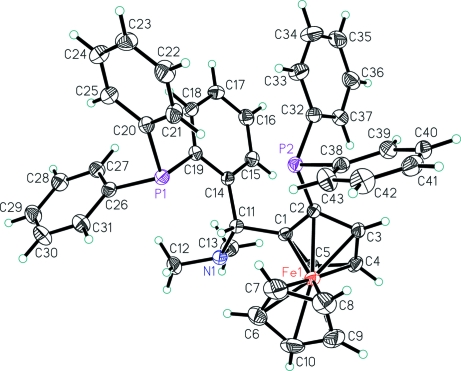
The structure of the title molecule, without the solvate group, shown with 50% probability displacement ellipsoids. The H atoms are drawn as small spheres of arbitrary radius.

## supplementary crystallographic information

### Comment

The Taniaphos ligand (Ireland *et al.*, 1999), a broadly used chiral ligand technology owned by Umicore and sold by Solvias, is a well known diphosphane ligand for catalytic asymmetric synthesis. Quite recently, the crystal structure of the solvent-free compound has been reported by Fukuzawa, Yamamoto & Kikuchi (2007). They showed that the planar chirality of the ligand formed by a classical *ortho*-directed metallation procedure (Fukuzawa, Yamamoto, Hosaka & Kikuchi, 2007) of a ferrocene with a chiral center configurated (*R*) in the side chain is (*R*_p_) and not (S_p_) as presumed previously. This was confirmed by comment of Ireland *et al.* (2008) on the crystal structure of a rhodium(I) complex of Taniaphos.

The molecular structure of the title compound is shown in Fig. 1. The absolute configuration of the molecule is *R*_p_ at the ferrocene group and *R* at the asymmetric carbon atom C11. The crystal structure of the solvent-free compound reported by Fukuzawa, Yamamoto & Kikuchi (2007) has two independent molecules in the asymmetric unit. The conformation of the three molecules is rather similar, but corresponding torsion angles about some *P*—C_phenyl_ bonds differ up to 25°.

The ferrocene group deviates 14° from an eclipsed conformation. The angle between the planes of the two cyclopentadienyl rings is 3.6 (1)°. Both P atoms have a pyramidal geometry with C—P—C angles between 99.7 (1) and 102.4 (1)°. The lone-pair lobe of atom P1 shows a short intramolecular contact distance of 2.57Å with the H atom of C11 (Table 1). There also is an intramolecular π···π contact with a distance of 3.333 (3)Å between C15 and C37. The N atom also has a pyramidal geometry. The N lone-pair is not involved in short intra- or intermolecular interactions. The crystal packing shows three intermolecular C—H···π_phenyl_ interactions (Table 1) with H···*Cg* distances shorter than 3Å (*Cg* represents the centroid of a phenyl ring). In one of them the chloroform C—H bond acts as a donor. There also are six intermolecular *C*—*H*···Cl_chloroform_ contacts with H···Cl distances between 2.96 and 3.13 Å.

### Experimental

Crystals were obtained by slow diffusion of diethyl ether into a chloroform solution of the commercially available Taniaphos ligand SL—T001–1. We have also performed a crystal structure determination of the commercially available Taniaphos ligand SL—T001–2, crystallized under similar conditions. The resulting crystal structure is enantiomorphous to the structure of the title compound. Thus the SL—T001–2 ligand has the S,*S*_p_ configuration.

### Refinement

H atoms were geometrically positioned using distances: C_planar_—H=0.95 Å, C_methyl_—H=0.98 Å, C_primary_—H=1.00 Å, *U*_iso_(*H*)=1.2*U*_eq_(C_non-methyl_) and *U*_iso_(*H*)=1.5*U*_eq_(C_methyl_). The torsion angles about the N—C bonds were varied for the methyl groups. Friedel opposites were not averaged. The absolute configuration was determined from 4811 Friedel pairs.

### Crystal data

**Table:**

[Fe(C_5_H_5_)(C_38_H_34_NP_2_)]·CHCl_3_	*F*_000_ = 1672
*M**_r_* = 806.91	*D*_x_ = 1.388 Mg m^−^^3^
Orthorhombic, *P*2_1_2_1_2_1_	Mo *K*α radiation λ = 0.71073 Å
Hall symbol: P 2ac 2ab	Cell parameters from 347 reflections
*a* = 10.6051 (11) Å	θ = 3–23º
*b* = 11.8922 (10) Å	µ = 0.71 mm^−^^1^
*c* = 30.625 (3) Å	*T* = 163 (2) K
*V* = 3862.3 (6) Å^3^	Block, yellow
*Z* = 4	0.60 × 0.40 × 0.37 mm

### Data collection

**Table:**

Siemens SMART 1K CCD diffractometer	11202 independent reflections
Radiation source: normal-focus sealed tube	9851 reflections with *I* > 2σ(*I*)
Monochromator: graphite	*R*_int_ = 0.061
*T* = 163(2) K	θ_max_ = 30.8º
ω scans	θ_min_ = 1.8º
Absorption correction: numerical(SHELXTL; Sheldrick, 2008)	*h* = −15→15
*T*_min_ = 0.664, *T*_max_ = 0.786	*k* = −16→16
59511 measured reflections	*l* = −43→44

### Refinement

**Table:**

Refinement on *F*^2^	Hydrogen site location: inferred from neighbouring sites
Least-squares matrix: full	H-atom parameters constrained
*R*[*F*^2^ > 2σ(*F*^2^)] = 0.039	*w* = 1/[σ^2^(*F*_o_^2^) + (0.03*P*)^2^ + 1.6*P*] where *P* = (*F*_o_^2^ + 2*F*_c_^2^)/3
*wR*(*F*^2^) = 0.088	(Δ/σ)_max_ = 0.003
*S* = 1.18	Δρ_max_ = 0.50 e Å^−^^3^
11202 reflections	Δρ_min_ = −0.39 e Å^−^^3^
462 parameters	Extinction correction: none
Primary atom site location: structure-invariant direct methods	Absolute structure: Flack (1983), 4811 Friedel pairs
Secondary atom site location: difference Fourier map	Flack parameter: −0.021 (11)

### Special details

**Table:**

Geometry. All e.s.d.'s (except the e.s.d. in the dihedral angle between two l.s. planes) are estimated using the full covariance matrix. The cell e.s.d.'s are taken into account individually in the estimation of e.s.d.'s in distances, angles and torsion angles; correlations between e.s.d.'s in cell parameters are only used when they are defined by crystal symmetry. An approximate (isotropic) treatment of cell e.s.d.'s is used for estimating e.s.d.'s involving l.s. planes.
Refinement. Refinement of *F*^2^ against ALL reflections. The weighted *R*-factor *wR* and goodness of fit *S* are based on *F*^2^, conventional *R*-factors *R* are based on *F*, with *F* set to zero for negative *F*^2^. The threshold expression of *F*^2^ > σ(*F*^2^) is used only for calculating *R*-factors(gt) *etc*. and is not relevant to the choice of reflections for refinement. *R*-factors based on *F*^2^ are statistically about twice as large as those based on *F*, and *R*- factors based on ALL data will be even larger.

### Fractional atomic coordinates and isotropic or equivalent isotropic displacement parameters (Å^2^)

**Table:**

	*x*	*y*	*z*	*U*_iso_*/*U*_eq_	
Fe1	0.74190 (3)	0.91368 (2)	0.919111 (9)	0.02233 (7)	
Cl1	0.95787 (8)	0.23821 (6)	0.77926 (3)	0.05234 (19)	
Cl2	1.06842 (7)	0.42876 (6)	0.73474 (2)	0.04212 (15)	
Cl3	1.16205 (7)	0.36270 (8)	0.81940 (3)	0.0571 (2)	
P1	0.70451 (5)	0.46648 (4)	0.915738 (17)	0.02029 (10)	
P2	0.53683 (5)	0.73476 (4)	0.867406 (17)	0.02087 (10)	
N1	0.98418 (17)	0.68727 (16)	0.88942 (6)	0.0253 (4)	
C1	0.79849 (19)	0.80313 (16)	0.87204 (6)	0.0190 (4)	
C2	0.66789 (19)	0.83355 (16)	0.86593 (7)	0.0197 (4)	
C3	0.6636 (2)	0.95373 (17)	0.85998 (7)	0.0217 (4)	
H3A	0.5897	0.9971	0.8552	0.026*	
C4	0.7886 (2)	0.99631 (17)	0.86250 (7)	0.0246 (4)	
H4A	0.8128	1.0729	0.8597	0.029*	
C5	0.87145 (19)	0.90374 (17)	0.86993 (7)	0.0228 (4)	
H5A	0.9605	0.9083	0.8730	0.027*	
C6	0.7954 (3)	0.8415 (2)	0.97653 (8)	0.0382 (6)	
H6A	0.8462	0.7761	0.9793	0.046*	
C7	0.6625 (3)	0.8439 (2)	0.97341 (8)	0.0388 (6)	
H7A	0.6083	0.7803	0.9739	0.047*	
C8	0.6239 (3)	0.9581 (2)	0.96934 (8)	0.0395 (6)	
H8A	0.5396	0.9840	0.9666	0.047*	
C9	0.7330 (3)	1.0258 (2)	0.97010 (8)	0.0419 (6)	
H9A	0.7350	1.1054	0.9679	0.050*	
C10	0.8396 (3)	0.9541 (2)	0.97474 (8)	0.0398 (6)	
H10A	0.9252	0.9774	0.9764	0.048*	
C11	0.84687 (19)	0.68498 (17)	0.88138 (6)	0.0200 (4)	
H11A	0.8070	0.6613	0.9095	0.024*	
C12	1.0266 (2)	0.59513 (19)	0.91705 (9)	0.0349 (5)	
H12A	1.1138	0.6088	0.9262	0.052*	
H12B	1.0224	0.5245	0.9007	0.052*	
H12C	0.9723	0.5901	0.9429	0.052*	
C13	1.0644 (2)	0.6962 (2)	0.85103 (8)	0.0326 (5)	
H13A	1.0602	0.6260	0.8343	0.049*	
H13B	1.1516	0.7099	0.8602	0.049*	
H13C	1.0353	0.7587	0.8327	0.049*	
C14	0.80052 (18)	0.60161 (16)	0.84674 (6)	0.0184 (4)	
C15	0.81624 (19)	0.62834 (18)	0.80262 (7)	0.0228 (4)	
H15A	0.8571	0.6966	0.7950	0.027*	
C16	0.7737 (2)	0.55772 (18)	0.76967 (7)	0.0258 (4)	
H16A	0.7859	0.5775	0.7399	0.031*	
C17	0.7132 (2)	0.45805 (18)	0.78045 (7)	0.0258 (4)	
H17A	0.6842	0.4089	0.7581	0.031*	
C18	0.6953 (2)	0.43071 (18)	0.82410 (7)	0.0232 (4)	
H18A	0.6527	0.3629	0.8312	0.028*	
C19	0.73836 (19)	0.50010 (16)	0.85791 (6)	0.0195 (4)	
C20	0.56331 (19)	0.37755 (17)	0.91069 (6)	0.0215 (4)	
C21	0.4471 (2)	0.43019 (18)	0.91673 (8)	0.0290 (4)	
H21A	0.4445	0.5084	0.9229	0.035*	
C22	0.3352 (2)	0.3702 (2)	0.91386 (9)	0.0355 (5)	
H22A	0.2566	0.4070	0.9183	0.043*	
C23	0.3388 (2)	0.2556 (2)	0.90449 (8)	0.0336 (5)	
H23A	0.2624	0.2144	0.9018	0.040*	
C24	0.4527 (2)	0.2020 (2)	0.89907 (8)	0.0295 (5)	
H24A	0.4546	0.1236	0.8932	0.035*	
C25	0.5648 (2)	0.26153 (18)	0.90216 (7)	0.0251 (4)	
H25A	0.6429	0.2236	0.8985	0.030*	
C26	0.82714 (19)	0.36238 (17)	0.92864 (7)	0.0219 (4)	
C27	0.8919 (2)	0.29840 (19)	0.89740 (7)	0.0255 (4)	
H27A	0.8704	0.3053	0.8674	0.031*	
C28	0.9872 (2)	0.22503 (19)	0.90990 (8)	0.0296 (5)	
H28A	1.0303	0.1817	0.8885	0.036*	
C29	1.0194 (2)	0.2150 (2)	0.95356 (8)	0.0337 (5)	
H29A	1.0854	0.1657	0.9621	0.040*	
C30	0.9560 (3)	0.2763 (2)	0.98461 (8)	0.0368 (5)	
H30A	0.9774	0.2681	1.0146	0.044*	
C31	0.8607 (2)	0.3506 (2)	0.97247 (8)	0.0297 (5)	
H31A	0.8182	0.3935	0.9941	0.036*	
C32	0.50410 (19)	0.71228 (17)	0.80926 (7)	0.0232 (4)	
C33	0.4184 (2)	0.6265 (2)	0.79871 (9)	0.0343 (5)	
H33A	0.3812	0.5828	0.8213	0.041*	
C34	0.3877 (3)	0.6052 (2)	0.75526 (10)	0.0446 (7)	
H34A	0.3282	0.5481	0.7484	0.054*	
C35	0.4428 (3)	0.6658 (2)	0.72231 (8)	0.0394 (6)	
H35A	0.4217	0.6503	0.6928	0.047*	
C36	0.5285 (2)	0.7493 (2)	0.73197 (8)	0.0335 (5)	
H36A	0.5671	0.7907	0.7091	0.040*	
C37	0.5589 (2)	0.77295 (19)	0.77527 (7)	0.0269 (4)	
H37A	0.6175	0.8311	0.7817	0.032*	
C38	0.4032 (2)	0.82758 (17)	0.88152 (7)	0.0232 (4)	
C39	0.3490 (2)	0.90539 (19)	0.85289 (7)	0.0268 (4)	
H39A	0.3821	0.9145	0.8243	0.032*	
C40	0.2458 (2)	0.96965 (18)	0.86647 (8)	0.0311 (5)	
H40A	0.2090	1.0221	0.8469	0.037*	
C41	0.1974 (2)	0.9581 (2)	0.90739 (9)	0.0356 (6)	
H41A	0.1284	1.0036	0.9163	0.043*	
C42	0.2483 (2)	0.8804 (2)	0.93601 (8)	0.0377 (5)	
H42A	0.2142	0.8719	0.9645	0.045*	
C43	0.3507 (2)	0.8141 (2)	0.92268 (8)	0.0320 (5)	
H43A	0.3845	0.7594	0.9420	0.038*	
C44	1.0275 (2)	0.3716 (2)	0.78598 (8)	0.0334 (5)	
H44A	0.9650	0.4226	0.8003	0.040*	

### Atomic displacement parameters (Å^2^)

**Table:**

	*U*^11^	*U*^22^	*U*^33^	*U*^12^	*U*^13^	*U*^23^
Fe1	0.02823 (14)	0.02016 (13)	0.01861 (13)	−0.00058 (12)	−0.00085 (12)	−0.00220 (11)
Cl1	0.0706 (5)	0.0357 (3)	0.0507 (4)	−0.0085 (3)	0.0253 (4)	−0.0095 (3)
Cl2	0.0541 (4)	0.0381 (3)	0.0341 (3)	−0.0010 (3)	0.0077 (3)	0.0013 (3)
Cl3	0.0390 (4)	0.0811 (6)	0.0512 (4)	0.0087 (4)	−0.0017 (3)	0.0119 (4)
P1	0.0234 (2)	0.0190 (2)	0.0185 (2)	−0.00097 (19)	0.00134 (19)	−0.0001 (2)
P2	0.0202 (2)	0.0194 (2)	0.0230 (2)	−0.0004 (2)	−0.00033 (19)	0.0015 (2)
N1	0.0227 (9)	0.0215 (8)	0.0316 (9)	−0.0020 (7)	−0.0068 (7)	0.0011 (8)
C1	0.0215 (9)	0.0176 (8)	0.0180 (9)	−0.0012 (7)	0.0002 (7)	−0.0020 (7)
C2	0.0222 (10)	0.0164 (9)	0.0204 (9)	−0.0007 (7)	−0.0007 (7)	0.0002 (7)
C3	0.0273 (10)	0.0186 (9)	0.0193 (9)	0.0014 (8)	−0.0017 (8)	0.0010 (7)
C4	0.0312 (11)	0.0168 (9)	0.0257 (10)	−0.0042 (8)	0.0012 (8)	0.0009 (8)
C5	0.0234 (9)	0.0211 (9)	0.0239 (10)	−0.0042 (8)	−0.0003 (7)	−0.0016 (8)
C6	0.0582 (16)	0.0380 (13)	0.0185 (10)	0.0055 (12)	−0.0065 (10)	0.0003 (9)
C7	0.0519 (15)	0.0478 (15)	0.0166 (10)	−0.0112 (13)	0.0064 (10)	0.0019 (10)
C8	0.0448 (15)	0.0508 (16)	0.0228 (11)	0.0083 (12)	0.0079 (10)	−0.0078 (11)
C9	0.0684 (19)	0.0326 (12)	0.0247 (11)	0.0027 (13)	−0.0013 (12)	−0.0111 (9)
C10	0.0492 (15)	0.0473 (15)	0.0229 (11)	−0.0112 (12)	−0.0081 (10)	−0.0067 (11)
C11	0.0201 (9)	0.0195 (9)	0.0203 (9)	−0.0005 (7)	−0.0017 (7)	−0.0004 (8)
C12	0.0297 (11)	0.0295 (11)	0.0456 (13)	0.0008 (9)	−0.0154 (11)	0.0083 (11)
C13	0.0205 (11)	0.0325 (11)	0.0447 (13)	−0.0007 (9)	0.0003 (9)	−0.0039 (10)
C14	0.0182 (8)	0.0171 (9)	0.0198 (9)	0.0007 (7)	−0.0007 (7)	0.0001 (7)
C15	0.0235 (10)	0.0212 (9)	0.0237 (10)	−0.0005 (8)	0.0016 (8)	0.0017 (8)
C16	0.0297 (11)	0.0286 (10)	0.0189 (9)	0.0027 (8)	0.0013 (8)	0.0016 (8)
C17	0.0292 (10)	0.0261 (10)	0.0220 (9)	−0.0017 (8)	−0.0034 (8)	−0.0037 (8)
C18	0.0263 (10)	0.0204 (10)	0.0230 (10)	−0.0028 (8)	−0.0001 (8)	−0.0007 (8)
C19	0.0188 (9)	0.0198 (8)	0.0199 (9)	0.0020 (7)	−0.0007 (7)	0.0014 (7)
C20	0.0230 (9)	0.0230 (9)	0.0186 (9)	−0.0014 (7)	0.0025 (7)	0.0021 (7)
C21	0.0288 (10)	0.0238 (10)	0.0343 (11)	0.0036 (8)	0.0008 (9)	0.0013 (9)
C22	0.0214 (10)	0.0358 (12)	0.0494 (15)	0.0070 (9)	0.0011 (10)	0.0010 (12)
C23	0.0230 (10)	0.0383 (13)	0.0396 (13)	−0.0071 (10)	−0.0020 (9)	0.0006 (10)
C24	0.0298 (11)	0.0264 (10)	0.0323 (11)	−0.0044 (9)	0.0033 (9)	−0.0027 (9)
C25	0.0248 (10)	0.0249 (10)	0.0256 (10)	−0.0005 (8)	0.0033 (8)	−0.0022 (8)
C26	0.0229 (9)	0.0198 (9)	0.0229 (10)	−0.0043 (7)	0.0007 (7)	0.0020 (7)
C27	0.0272 (11)	0.0269 (10)	0.0225 (10)	−0.0001 (8)	−0.0001 (8)	0.0003 (8)
C28	0.0250 (10)	0.0289 (11)	0.0349 (12)	0.0019 (9)	0.0021 (9)	−0.0001 (9)
C29	0.0265 (11)	0.0344 (13)	0.0400 (13)	0.0031 (10)	−0.0068 (10)	0.0059 (10)
C30	0.0401 (13)	0.0448 (14)	0.0254 (11)	0.0045 (12)	−0.0081 (10)	0.0059 (10)
C31	0.0298 (11)	0.0350 (12)	0.0243 (11)	0.0005 (9)	−0.0013 (9)	0.0031 (9)
C32	0.0208 (9)	0.0224 (10)	0.0263 (10)	0.0014 (8)	−0.0021 (8)	−0.0037 (8)
C33	0.0334 (12)	0.0294 (11)	0.0402 (13)	−0.0075 (10)	−0.0029 (10)	−0.0029 (10)
C34	0.0460 (15)	0.0355 (14)	0.0524 (17)	−0.0076 (12)	−0.0146 (13)	−0.0137 (12)
C35	0.0511 (16)	0.0360 (13)	0.0310 (12)	0.0098 (12)	−0.0162 (11)	−0.0105 (10)
C36	0.0355 (12)	0.0387 (13)	0.0261 (11)	0.0057 (10)	−0.0041 (9)	−0.0012 (10)
C37	0.0251 (10)	0.0276 (10)	0.0279 (11)	0.0010 (9)	−0.0039 (8)	0.0001 (9)
C38	0.0196 (9)	0.0226 (10)	0.0273 (10)	−0.0010 (8)	−0.0002 (8)	−0.0033 (8)
C39	0.0244 (9)	0.0273 (10)	0.0288 (10)	0.0002 (8)	−0.0014 (8)	−0.0001 (9)
C40	0.0274 (10)	0.0227 (10)	0.0432 (12)	0.0032 (9)	−0.0100 (10)	−0.0012 (9)
C41	0.0235 (10)	0.0307 (11)	0.0526 (16)	0.0028 (9)	0.0006 (10)	−0.0118 (11)
C42	0.0324 (12)	0.0464 (14)	0.0344 (12)	0.0011 (12)	0.0123 (10)	0.0000 (10)
C43	0.0235 (10)	0.0410 (12)	0.0314 (12)	0.0041 (9)	0.0045 (9)	0.0073 (11)
C44	0.0357 (12)	0.0342 (12)	0.0302 (12)	0.0036 (10)	0.0067 (10)	−0.0018 (9)

### Geometric parameters (Å, °)

**Table:**

Fe1—C6	2.037 (2)	C15—H15A	0.9500
Fe1—C7	2.040 (2)	C16—C17	1.388 (3)
Fe1—C1	2.041 (2)	C16—H16A	0.9500
Fe1—C5	2.042 (2)	C17—C18	1.389 (3)
Fe1—C2	2.044 (2)	C17—H17A	0.9500
Fe1—C3	2.048 (2)	C18—C19	1.400 (3)
Fe1—C10	2.051 (2)	C18—H18A	0.9500
Fe1—C8	2.052 (2)	C20—C21	1.395 (3)
Fe1—C4	2.053 (2)	C20—C25	1.404 (3)
Fe1—C9	2.055 (2)	C21—C22	1.388 (3)
Cl1—C44	1.762 (3)	C21—H21A	0.9500
Cl2—C44	1.765 (2)	C22—C23	1.393 (4)
Cl3—C44	1.759 (3)	C22—H22A	0.9500
P1—C26	1.839 (2)	C23—C24	1.376 (3)
P1—C20	1.840 (2)	C23—H23A	0.9500
P1—C19	1.851 (2)	C24—C25	1.386 (3)
P2—C2	1.820 (2)	C24—H24A	0.9500
P2—C32	1.834 (2)	C25—H25A	0.9500
P2—C38	1.847 (2)	C26—C31	1.395 (3)
N1—C13	1.455 (3)	C26—C27	1.402 (3)
N1—C12	1.456 (3)	C27—C28	1.389 (3)
N1—C11	1.477 (3)	C27—H27A	0.9500
C1—C5	1.426 (3)	C28—C29	1.385 (3)
C1—C2	1.444 (3)	C28—H28A	0.9500
C1—C11	1.523 (3)	C29—C30	1.374 (4)
C2—C3	1.441 (3)	C29—H29A	0.9500
C3—C4	1.421 (3)	C30—C31	1.394 (3)
C3—H3A	0.9500	C30—H30A	0.9500
C4—C5	1.427 (3)	C31—H31A	0.9500
C4—H4A	0.9500	C32—C37	1.393 (3)
C5—H5A	0.9500	C32—C33	1.404 (3)
C6—C7	1.413 (4)	C33—C34	1.393 (4)
C6—C10	1.420 (4)	C33—H33A	0.9500
C6—H6A	0.9500	C34—C35	1.371 (4)
C7—C8	1.423 (4)	C34—H34A	0.9500
C7—H7A	0.9500	C35—C36	1.378 (4)
C8—C9	1.410 (4)	C35—H35A	0.9500
C8—H8A	0.9500	C36—C37	1.393 (3)
C9—C10	1.423 (4)	C36—H36A	0.9500
C9—H9A	0.9500	C37—H37A	0.9500
C10—H10A	0.9500	C38—C43	1.388 (3)
C11—C14	1.533 (3)	C38—C39	1.399 (3)
C11—H11A	1.0000	C39—C40	1.398 (3)
C12—H12A	0.9800	C39—H39A	0.9500
C12—H12B	0.9800	C40—C41	1.361 (4)
C12—H12C	0.9800	C40—H40A	0.9500
C13—H13A	0.9800	C41—C42	1.384 (4)
C13—H13B	0.9800	C41—H41A	0.9500
C13—H13C	0.9800	C42—C43	1.402 (3)
C14—C15	1.398 (3)	C42—H42A	0.9500
C14—C19	1.417 (3)	C43—H43A	0.9500
C15—C16	1.388 (3)	C44—H44A	1.0000
			
C6—Fe1—C7	40.54 (12)	N1—C11—C1	110.27 (17)
C6—Fe1—C1	104.86 (10)	N1—C11—C14	116.32 (17)
C7—Fe1—C1	115.79 (10)	C1—C11—C14	111.02 (16)
C6—Fe1—C5	115.15 (10)	N1—C11—H11A	106.2
C7—Fe1—C5	148.79 (11)	C1—C11—H11A	106.2
C1—Fe1—C5	40.89 (8)	C14—C11—H11A	106.2
C6—Fe1—C2	126.76 (10)	N1—C12—H12A	109.5
C7—Fe1—C2	107.55 (9)	N1—C12—H12B	109.5
C1—Fe1—C2	41.39 (8)	H12A—C12—H12B	109.5
C5—Fe1—C2	69.12 (8)	N1—C12—H12C	109.5
C6—Fe1—C3	166.89 (10)	H12A—C12—H12C	109.5
C7—Fe1—C3	130.39 (11)	H12B—C12—H12C	109.5
C1—Fe1—C3	69.17 (8)	N1—C13—H13A	109.5
C5—Fe1—C3	68.53 (8)	N1—C13—H13B	109.5
C2—Fe1—C3	41.25 (8)	H13A—C13—H13B	109.5
C6—Fe1—C10	40.63 (11)	N1—C13—H13C	109.5
C7—Fe1—C10	68.09 (11)	H13A—C13—H13C	109.5
C1—Fe1—C10	126.08 (11)	H13B—C13—H13C	109.5
C5—Fe1—C10	106.63 (10)	C15—C14—C19	118.85 (18)
C2—Fe1—C10	164.84 (10)	C15—C14—C11	118.92 (17)
C3—Fe1—C10	152.20 (10)	C19—C14—C11	122.20 (17)
C6—Fe1—C8	68.35 (12)	C16—C15—C14	121.75 (19)
C7—Fe1—C8	40.70 (12)	C16—C15—H15A	119.1
C1—Fe1—C8	150.97 (10)	C14—C15—H15A	119.1
C5—Fe1—C8	167.98 (11)	C17—C16—C15	119.61 (19)
C2—Fe1—C8	118.89 (10)	C17—C16—H16A	120.2
C3—Fe1—C8	110.85 (10)	C15—C16—H16A	120.2
C10—Fe1—C8	67.99 (12)	C16—C17—C18	119.5 (2)
C6—Fe1—C4	149.67 (11)	C16—C17—H17A	120.3
C7—Fe1—C4	169.20 (11)	C18—C17—H17A	120.3
C1—Fe1—C4	68.96 (8)	C17—C18—C19	121.97 (19)
C5—Fe1—C4	40.77 (8)	C17—C18—H18A	119.0
C2—Fe1—C4	69.08 (8)	C19—C18—H18A	119.0
C3—Fe1—C4	40.54 (8)	C18—C19—C14	118.35 (18)
C10—Fe1—C4	117.85 (10)	C18—C19—P1	121.13 (15)
C8—Fe1—C4	131.07 (10)	C14—C19—P1	120.35 (14)
C6—Fe1—C9	68.30 (11)	C21—C20—C25	118.39 (19)
C7—Fe1—C9	68.00 (11)	C21—C20—P1	116.76 (16)
C1—Fe1—C9	165.43 (11)	C25—C20—P1	124.84 (16)
C5—Fe1—C9	128.97 (11)	C22—C21—C20	121.1 (2)
C2—Fe1—C9	152.90 (11)	C22—C21—H21A	119.4
C3—Fe1—C9	120.15 (10)	C20—C21—H21A	119.4
C10—Fe1—C9	40.56 (12)	C21—C22—C23	119.5 (2)
C8—Fe1—C9	40.14 (12)	C21—C22—H22A	120.2
C4—Fe1—C9	110.01 (10)	C23—C22—H22A	120.2
C26—P1—C20	101.93 (9)	C24—C23—C22	120.1 (2)
C26—P1—C19	102.35 (9)	C24—C23—H23A	119.9
C20—P1—C19	101.64 (9)	C22—C23—H23A	119.9
C2—P2—C32	102.38 (10)	C23—C24—C25	120.6 (2)
C2—P2—C38	101.87 (9)	C23—C24—H24A	119.7
C32—P2—C38	99.73 (10)	C25—C24—H24A	119.7
C13—N1—C12	110.10 (19)	C24—C25—C20	120.3 (2)
C13—N1—C11	116.29 (18)	C24—C25—H25A	119.9
C12—N1—C11	112.82 (18)	C20—C25—H25A	119.9
C5—C1—C2	107.72 (17)	C31—C26—C27	118.5 (2)
C5—C1—C11	126.85 (18)	C31—C26—P1	117.03 (17)
C2—C1—C11	125.36 (17)	C27—C26—P1	124.42 (16)
C5—C1—Fe1	69.58 (11)	C28—C27—C26	120.6 (2)
C2—C1—Fe1	69.40 (11)	C28—C27—H27A	119.7
C11—C1—Fe1	124.09 (14)	C26—C27—H27A	119.7
C3—C2—C1	107.15 (18)	C29—C28—C27	119.9 (2)
C3—C2—P2	128.26 (16)	C29—C28—H28A	120.0
C1—C2—P2	124.57 (14)	C27—C28—H28A	120.0
C3—C2—Fe1	69.54 (12)	C30—C29—C28	120.2 (2)
C1—C2—Fe1	69.21 (11)	C30—C29—H29A	119.9
P2—C2—Fe1	125.05 (11)	C28—C29—H29A	119.9
C4—C3—C2	108.50 (18)	C29—C30—C31	120.4 (2)
C4—C3—Fe1	69.92 (12)	C29—C30—H30A	119.8
C2—C3—Fe1	69.21 (12)	C31—C30—H30A	119.8
C4—C3—H3A	125.7	C30—C31—C26	120.3 (2)
C2—C3—H3A	125.7	C30—C31—H31A	119.8
Fe1—C3—H3A	126.7	C26—C31—H31A	119.8
C3—C4—C5	107.94 (17)	C37—C32—C33	118.3 (2)
C3—C4—Fe1	69.54 (12)	C37—C32—P2	124.82 (16)
C5—C4—Fe1	69.19 (12)	C33—C32—P2	116.86 (18)
C3—C4—H4A	126.0	C34—C33—C32	120.2 (2)
C5—C4—H4A	126.0	C34—C33—H33A	119.9
Fe1—C4—H4A	126.8	C32—C33—H33A	119.9
C1—C5—C4	108.69 (17)	C35—C34—C33	120.5 (2)
C1—C5—Fe1	69.53 (11)	C35—C34—H34A	119.7
C4—C5—Fe1	70.04 (12)	C33—C34—H34A	119.7
C1—C5—H5A	125.7	C34—C35—C36	120.1 (2)
C4—C5—H5A	125.7	C34—C35—H35A	119.9
Fe1—C5—H5A	126.4	C36—C35—H35A	119.9
C7—C6—C10	107.9 (3)	C35—C36—C37	120.1 (2)
C7—C6—Fe1	69.84 (15)	C35—C36—H36A	119.9
C10—C6—Fe1	70.21 (15)	C37—C36—H36A	119.9
C7—C6—H6A	126.0	C32—C37—C36	120.7 (2)
C10—C6—H6A	126.0	C32—C37—H37A	119.7
Fe1—C6—H6A	125.5	C36—C37—H37A	119.7
C6—C7—C8	108.2 (3)	C43—C38—C39	118.7 (2)
C6—C7—Fe1	69.61 (15)	C43—C38—P2	116.83 (17)
C8—C7—Fe1	70.10 (15)	C39—C38—P2	124.35 (17)
C6—C7—H7A	125.9	C40—C39—C38	119.8 (2)
C8—C7—H7A	125.9	C40—C39—H39A	120.1
Fe1—C7—H7A	126.0	C38—C39—H39A	120.1
C9—C8—C7	107.9 (3)	C41—C40—C39	120.9 (2)
C9—C8—Fe1	70.05 (14)	C41—C40—H40A	119.5
C7—C8—Fe1	69.20 (14)	C39—C40—H40A	119.5
C9—C8—H8A	126.1	C40—C41—C42	120.2 (2)
C7—C8—H8A	126.1	C40—C41—H41A	119.9
Fe1—C8—H8A	126.3	C42—C41—H41A	119.9
C8—C9—C10	108.2 (2)	C41—C42—C43	119.6 (2)
C8—C9—Fe1	69.81 (14)	C41—C42—H42A	120.2
C10—C9—Fe1	69.57 (14)	C43—C42—H42A	120.2
C8—C9—H9A	125.9	C38—C43—C42	120.7 (2)
C10—C9—H9A	125.9	C38—C43—H43A	119.7
Fe1—C9—H9A	126.3	C42—C43—H43A	119.7
C6—C10—C9	107.8 (3)	Cl3—C44—Cl1	110.73 (14)
C6—C10—Fe1	69.15 (14)	Cl3—C44—Cl2	109.94 (14)
C9—C10—Fe1	69.87 (14)	Cl1—C44—Cl2	110.26 (14)
C6—C10—H10A	126.1	Cl3—C44—H44A	108.6
C9—C10—H10A	126.1	Cl1—C44—H44A	108.6
Fe1—C10—H10A	126.5	Cl2—C44—H44A	108.6
			
C6—Fe1—C1—C5	−111.42 (14)	C3—Fe1—C7—C6	166.56 (14)
C7—Fe1—C1—C5	−153.26 (14)	C10—Fe1—C7—C6	−37.99 (17)
C2—Fe1—C1—C5	119.14 (16)	C8—Fe1—C7—C6	−119.3 (2)
C3—Fe1—C1—C5	80.85 (13)	C4—Fe1—C7—C6	−163.4 (5)
C10—Fe1—C1—C5	−72.51 (16)	C9—Fe1—C7—C6	−81.88 (18)
C8—Fe1—C1—C5	176.55 (19)	C6—Fe1—C7—C8	119.3 (2)
C4—Fe1—C1—C5	37.31 (12)	C1—Fe1—C7—C8	−158.02 (15)
C9—Fe1—C1—C5	−51.4 (4)	C5—Fe1—C7—C8	167.33 (17)
C6—Fe1—C1—C2	129.45 (13)	C2—Fe1—C7—C8	−114.17 (17)
C7—Fe1—C1—C2	87.60 (14)	C3—Fe1—C7—C8	−74.2 (2)
C5—Fe1—C1—C2	−119.14 (16)	C10—Fe1—C7—C8	81.27 (18)
C3—Fe1—C1—C2	−38.28 (12)	C4—Fe1—C7—C8	−44.1 (6)
C10—Fe1—C1—C2	168.36 (13)	C9—Fe1—C7—C8	37.38 (17)
C8—Fe1—C1—C2	57.4 (2)	C6—C7—C8—C9	−0.2 (3)
C4—Fe1—C1—C2	−81.82 (12)	Fe1—C7—C8—C9	−59.59 (17)
C9—Fe1—C1—C2	−170.5 (4)	C6—C7—C8—Fe1	59.41 (18)
C6—Fe1—C1—C11	9.96 (19)	C6—Fe1—C8—C9	81.58 (18)
C7—Fe1—C1—C11	−31.9 (2)	C7—Fe1—C8—C9	119.2 (2)
C5—Fe1—C1—C11	121.4 (2)	C1—Fe1—C8—C9	163.15 (18)
C2—Fe1—C1—C11	−119.5 (2)	C5—Fe1—C8—C9	−27.8 (6)
C3—Fe1—C1—C11	−157.77 (18)	C2—Fe1—C8—C9	−157.33 (15)
C10—Fe1—C1—C11	48.9 (2)	C3—Fe1—C8—C9	−112.47 (16)
C8—Fe1—C1—C11	−62.1 (3)	C10—Fe1—C8—C9	37.65 (16)
C4—Fe1—C1—C11	158.69 (19)	C4—Fe1—C8—C9	−70.8 (2)
C9—Fe1—C1—C11	70.0 (4)	C6—Fe1—C8—C7	−37.60 (17)
C5—C1—C2—C3	0.2 (2)	C1—Fe1—C8—C7	44.0 (3)
C11—C1—C2—C3	177.32 (18)	C5—Fe1—C8—C7	−146.9 (4)
Fe1—C1—C2—C3	59.44 (14)	C2—Fe1—C8—C7	83.49 (18)
C5—C1—C2—P2	−178.22 (15)	C3—Fe1—C8—C7	128.36 (16)
C11—C1—C2—P2	−1.1 (3)	C10—Fe1—C8—C7	−81.53 (18)
Fe1—C1—C2—P2	−118.97 (16)	C4—Fe1—C8—C7	170.04 (15)
C5—C1—C2—Fe1	−59.24 (14)	C9—Fe1—C8—C7	−119.2 (2)
C11—C1—C2—Fe1	117.87 (19)	C7—C8—C9—C10	−0.1 (3)
C32—P2—C2—C3	81.2 (2)	Fe1—C8—C9—C10	−59.20 (17)
C38—P2—C2—C3	−21.7 (2)	C7—C8—C9—Fe1	59.06 (17)
C32—P2—C2—C1	−100.70 (18)	C6—Fe1—C9—C8	−81.72 (18)
C38—P2—C2—C1	156.42 (18)	C7—Fe1—C9—C8	−37.89 (17)
C32—P2—C2—Fe1	171.77 (12)	C1—Fe1—C9—C8	−146.0 (4)
C38—P2—C2—Fe1	68.89 (14)	C5—Fe1—C9—C8	172.83 (15)
C6—Fe1—C2—C3	172.74 (15)	C2—Fe1—C9—C8	47.8 (3)
C7—Fe1—C2—C3	132.08 (15)	C3—Fe1—C9—C8	87.11 (18)
C1—Fe1—C2—C3	−118.57 (17)	C10—Fe1—C9—C8	−119.4 (2)
C5—Fe1—C2—C3	−80.84 (13)	C4—Fe1—C9—C8	130.74 (16)
C10—Fe1—C2—C3	−157.2 (4)	C6—Fe1—C9—C10	37.71 (17)
C8—Fe1—C2—C3	89.27 (15)	C7—Fe1—C9—C10	81.55 (18)
C4—Fe1—C2—C3	−37.05 (13)	C1—Fe1—C9—C10	−26.6 (5)
C9—Fe1—C2—C3	56.2 (3)	C5—Fe1—C9—C10	−67.73 (19)
C6—Fe1—C2—C1	−68.69 (16)	C2—Fe1—C9—C10	167.21 (19)
C7—Fe1—C2—C1	−109.35 (14)	C3—Fe1—C9—C10	−153.45 (15)
C5—Fe1—C2—C1	37.73 (11)	C8—Fe1—C9—C10	119.4 (2)
C3—Fe1—C2—C1	118.57 (17)	C4—Fe1—C9—C10	−109.82 (16)
C10—Fe1—C2—C1	−38.6 (4)	C7—C6—C10—C9	−0.5 (3)
C8—Fe1—C2—C1	−152.16 (13)	Fe1—C6—C10—C9	59.39 (17)
C4—Fe1—C2—C1	81.52 (12)	C7—C6—C10—Fe1	−59.91 (18)
C9—Fe1—C2—C1	174.8 (2)	C8—C9—C10—C6	0.4 (3)
C6—Fe1—C2—P2	49.67 (18)	Fe1—C9—C10—C6	−58.94 (17)
C7—Fe1—C2—P2	9.02 (16)	C8—C9—C10—Fe1	59.35 (16)
C1—Fe1—C2—P2	118.37 (17)	C7—Fe1—C10—C6	37.91 (18)
C5—Fe1—C2—P2	156.10 (15)	C1—Fe1—C10—C6	−68.8 (2)
C3—Fe1—C2—P2	−123.06 (19)	C5—Fe1—C10—C6	−109.44 (17)
C10—Fe1—C2—P2	79.8 (4)	C2—Fe1—C10—C6	−38.1 (5)
C8—Fe1—C2—P2	−33.79 (17)	C3—Fe1—C10—C6	175.2 (2)
C4—Fe1—C2—P2	−160.12 (15)	C8—Fe1—C10—C6	81.96 (18)
C9—Fe1—C2—P2	−66.8 (3)	C4—Fe1—C10—C6	−152.04 (16)
C1—C2—C3—C4	−0.2 (2)	C9—Fe1—C10—C6	119.2 (2)
P2—C2—C3—C4	178.16 (16)	C6—Fe1—C10—C9	−119.2 (2)
Fe1—C2—C3—C4	59.05 (15)	C7—Fe1—C10—C9	−81.32 (19)
C1—C2—C3—Fe1	−59.23 (14)	C1—Fe1—C10—C9	171.99 (15)
P2—C2—C3—Fe1	119.10 (18)	C5—Fe1—C10—C9	131.33 (16)
C6—Fe1—C3—C4	−146.5 (4)	C2—Fe1—C10—C9	−157.3 (3)
C7—Fe1—C3—C4	171.69 (14)	C3—Fe1—C10—C9	56.0 (3)
C1—Fe1—C3—C4	−81.60 (13)	C8—Fe1—C10—C9	−37.27 (17)
C5—Fe1—C3—C4	−37.61 (12)	C4—Fe1—C10—C9	88.73 (18)
C2—Fe1—C3—C4	−120.01 (17)	C13—N1—C11—C1	−78.0 (2)
C10—Fe1—C3—C4	47.4 (3)	C12—N1—C11—C1	153.44 (19)
C8—Fe1—C3—C4	129.51 (14)	C13—N1—C11—C14	49.6 (3)
C9—Fe1—C3—C4	85.97 (16)	C12—N1—C11—C14	−79.0 (2)
C6—Fe1—C3—C2	−26.5 (5)	C5—C1—C11—N1	1.4 (3)
C7—Fe1—C3—C2	−68.30 (17)	C2—C1—C11—N1	−175.17 (19)
C1—Fe1—C3—C2	38.41 (12)	Fe1—C1—C11—N1	−87.5 (2)
C5—Fe1—C3—C2	82.40 (13)	C5—C1—C11—C14	−129.0 (2)
C10—Fe1—C3—C2	167.4 (2)	C2—C1—C11—C14	54.4 (3)
C8—Fe1—C3—C2	−110.48 (14)	Fe1—C1—C11—C14	142.10 (15)
C4—Fe1—C3—C2	120.01 (17)	N1—C11—C14—C15	−77.1 (2)
C9—Fe1—C3—C2	−154.02 (14)	C1—C11—C14—C15	50.1 (2)
C2—C3—C4—C5	0.1 (2)	N1—C11—C14—C19	105.1 (2)
Fe1—C3—C4—C5	58.71 (15)	C1—C11—C14—C19	−127.73 (19)
C2—C3—C4—Fe1	−58.62 (14)	C19—C14—C15—C16	−0.6 (3)
C6—Fe1—C4—C3	165.66 (18)	C11—C14—C15—C16	−178.46 (19)
C7—Fe1—C4—C3	−36.0 (6)	C14—C15—C16—C17	0.4 (3)
C1—Fe1—C4—C3	82.16 (12)	C15—C16—C17—C18	0.4 (3)
C5—Fe1—C4—C3	119.58 (17)	C16—C17—C18—C19	−1.0 (3)
C2—Fe1—C4—C3	37.68 (12)	C17—C18—C19—C14	0.7 (3)
C10—Fe1—C4—C3	−157.14 (14)	C17—C18—C19—P1	176.02 (17)
C8—Fe1—C4—C3	−72.99 (17)	C15—C14—C19—C18	0.1 (3)
C9—Fe1—C4—C3	−113.37 (14)	C11—C14—C19—C18	177.84 (18)
C6—Fe1—C4—C5	46.1 (2)	C15—C14—C19—P1	−175.28 (15)
C7—Fe1—C4—C5	−155.6 (5)	C11—C14—C19—P1	2.5 (3)
C1—Fe1—C4—C5	−37.42 (12)	C26—P1—C19—C18	81.89 (18)
C2—Fe1—C4—C5	−81.90 (13)	C20—P1—C19—C18	−23.25 (19)
C3—Fe1—C4—C5	−119.58 (17)	C26—P1—C19—C14	−102.91 (17)
C10—Fe1—C4—C5	83.28 (15)	C20—P1—C19—C14	151.95 (16)
C8—Fe1—C4—C5	167.43 (14)	C26—P1—C20—C21	157.26 (17)
C9—Fe1—C4—C5	127.05 (14)	C19—P1—C20—C21	−97.27 (17)
C2—C1—C5—C4	−0.1 (2)	C26—P1—C20—C25	−21.4 (2)
C11—C1—C5—C4	−177.21 (19)	C19—P1—C20—C25	84.06 (19)
Fe1—C1—C5—C4	−59.27 (15)	C25—C20—C21—C22	−0.8 (3)
C2—C1—C5—Fe1	59.13 (14)	P1—C20—C21—C22	−179.6 (2)
C11—C1—C5—Fe1	−117.9 (2)	C20—C21—C22—C23	−0.4 (4)
C3—C4—C5—C1	0.0 (2)	C21—C22—C23—C24	1.4 (4)
Fe1—C4—C5—C1	58.96 (14)	C22—C23—C24—C25	−1.0 (4)
C3—C4—C5—Fe1	−58.92 (14)	C23—C24—C25—C20	−0.3 (3)
C6—Fe1—C5—C1	83.73 (14)	C21—C20—C25—C24	1.2 (3)
C7—Fe1—C5—C1	51.4 (2)	P1—C20—C25—C24	179.85 (17)
C2—Fe1—C5—C1	−38.18 (11)	C20—P1—C26—C31	−99.02 (18)
C3—Fe1—C5—C1	−82.56 (13)	C19—P1—C26—C31	156.06 (17)
C10—Fe1—C5—C1	126.44 (13)	C20—P1—C26—C27	83.79 (19)
C8—Fe1—C5—C1	−171.9 (4)	C19—P1—C26—C27	−21.1 (2)
C4—Fe1—C5—C1	−119.97 (17)	C31—C26—C27—C28	0.0 (3)
C9—Fe1—C5—C1	165.35 (14)	P1—C26—C27—C28	177.18 (17)
C6—Fe1—C5—C4	−156.31 (13)	C26—C27—C28—C29	−0.3 (3)
C7—Fe1—C5—C4	171.40 (18)	C27—C28—C29—C30	0.9 (4)
C1—Fe1—C5—C4	119.97 (17)	C28—C29—C30—C31	−1.2 (4)
C2—Fe1—C5—C4	81.79 (13)	C29—C30—C31—C26	0.9 (4)
C3—Fe1—C5—C4	37.40 (12)	C27—C26—C31—C30	−0.3 (3)
C10—Fe1—C5—C4	−113.59 (14)	P1—C26—C31—C30	−177.69 (19)
C8—Fe1—C5—C4	−52.0 (5)	C2—P2—C32—C37	−8.3 (2)
C9—Fe1—C5—C4	−74.69 (17)	C38—P2—C32—C37	96.3 (2)
C1—Fe1—C6—C7	−112.48 (17)	C2—P2—C32—C33	170.98 (17)
C5—Fe1—C6—C7	−154.79 (16)	C38—P2—C32—C33	−84.46 (19)
C2—Fe1—C6—C7	−72.88 (19)	C37—C32—C33—C34	−1.4 (4)
C3—Fe1—C6—C7	−51.3 (5)	P2—C32—C33—C34	179.3 (2)
C10—Fe1—C6—C7	118.7 (2)	C32—C33—C34—C35	1.4 (4)
C8—Fe1—C6—C7	37.74 (17)	C33—C34—C35—C36	−0.5 (4)
C4—Fe1—C6—C7	173.90 (17)	C34—C35—C36—C37	−0.5 (4)
C9—Fe1—C6—C7	81.09 (19)	C33—C32—C37—C36	0.4 (3)
C7—Fe1—C6—C10	−118.7 (2)	P2—C32—C37—C36	179.62 (18)
C1—Fe1—C6—C10	128.79 (17)	C35—C36—C37—C32	0.6 (4)
C5—Fe1—C6—C10	86.48 (18)	C2—P2—C38—C43	−110.84 (19)
C2—Fe1—C6—C10	168.38 (16)	C32—P2—C38—C43	144.19 (18)
C3—Fe1—C6—C10	−170.1 (4)	C2—P2—C38—C39	72.4 (2)
C8—Fe1—C6—C10	−80.99 (18)	C32—P2—C38—C39	−32.6 (2)
C4—Fe1—C6—C10	55.2 (3)	C43—C38—C39—C40	1.6 (3)
C9—Fe1—C6—C10	−37.64 (18)	P2—C38—C39—C40	178.31 (17)
C10—C6—C7—C8	0.4 (3)	C38—C39—C40—C41	0.3 (3)
Fe1—C6—C7—C8	−59.71 (18)	C39—C40—C41—C42	−1.4 (4)
C10—C6—C7—Fe1	60.14 (18)	C40—C41—C42—C43	0.5 (4)
C1—Fe1—C7—C6	82.72 (17)	C39—C38—C43—C42	−2.5 (4)
C5—Fe1—C7—C6	48.1 (3)	P2—C38—C43—C42	−179.47 (19)
C2—Fe1—C7—C6	126.58 (16)	C41—C42—C43—C38	1.5 (4)

### Hydrogen-bond geometry (Å, °)

**Table:**

*D*—H···*A*	*D*—H	H···*A*	*D*···*A*	*D*—H···*A*
C11—H11A···P1	1.00	2.57	3.184 (2)	120
C13—H13B···Cg(C38->C43)^i^	0.98	2.88	3.663	138
C30—H30A···Cg(C20->C25)^ii^	0.95	2.59	3.473	154
C44—H44A···Cg(C14->C19)	1.00	2.59	3.537	159

Symmetry codes:  (i) *x*+1, *y*, *z*; (ii) *x*+1/2, −*y*+1/2, −*z*+2.
